# Impact of seasonal and meteorological factors on the incidence of adhesive small bowel obstruction: A large‐scale study using a national inpatient database

**DOI:** 10.1002/ags3.12541

**Published:** 2021-12-28

**Authors:** Yuta Yamamoto, Masato Kitazawa, Tetsuya Otsubo, Yusuke Miyagawa, Shigeo Tokumaru, Satoshi Nakamura, Makoto Koyama, Takehito Ehara, Nao Hondo, Yuji Soejima

**Affiliations:** ^1^ Division of Gastroenterological Hepato‐Biliary‐Pancreatic, Transplantation and Pediatric Surgery Department of Surgery Shinshu University School of Medicine Matsumoto, Nagano Japan; ^2^ The Database Center of the National University Hospitals The University of Tokyo Hospital Tokyo Japan; ^3^ Division of Medical Information Technology and Administration Planning Kyoto University Hospital Kyoto Japan

**Keywords:** adhesive small bowel obstruction, admission, barometric pressure, humidity, seasonal variation

## Abstract

**Aim:**

Whether seasonal and meteorological factors affect the incidence of adhesive small bowel obstruction (ASBO) remains unclear. This study aimed to clarify the impacts of seasonal and meteorological factors on the occurrence of ASBO.

**Methods:**

Clinical data of patients with ASBO were acquired from 42 national university hospitals in Japan, using a national inpatient database, between April 2012 and March 2020. Meteorological data were obtained from the Japan Meteorological Agency. The number of monthly admissions of patients with ASBO was compared between each of the 12 months. Daily weather variables were investigated to clarify their association with ASBO patient admissions on a total of 119 802 days (Formula for calculation: study period [2922 days] ×41 cities).

**Results:**

Overall, 4985 patients with ASBO were admitted. The number of admissions in June was smaller than that in October, November, and December (39 vs 63.5, *P* = .002, 39 vs 65, *P* = .004, and 39 vs 59.5, *P* = .002, respectively). Logistic regression analysis revealed that January, October, November, and December were associated with increased risk of admission compared to June (odds ratio [OR], 1.264; *P* = .001; OR, 1.454; *P* < .001; OR, 1.408; *P* < .001; OR, 1.330; *P* < .001), respectively. Regarding the weather variables, higher barometric pressure and lower humidity were associated with increased risk of admission (OR, 1.011; *P* < .001 and OR, 0.995; *P* < .001), respectively.

**Conclusion:**

The incidence of ASBO is susceptible to barometric pressure and humidity and varies monthly. These results can contribute to the prevention, early detection, and immediate and appropriate management of ASBO.

## Graphical abstract

The incidence of adhesive small bowel obstruction varies from month to month. Higher barometric pressure and lower humidity were associated with an increased risk of admissions of adhesive small bowel obstruction.

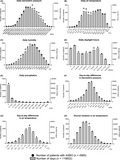

## 1 BACKGROUND

Adhesive small bowel obstruction (ASBO) is a common disorder of the small intestine. Although the risk of ASBO is decreasing due to the expansion of laparoscopic surgery, patients with this problem comprise a substantial percentage of admissions after intra‐abdominal surgery.^1^ Appropriate and prompt responses, including surgical intervention, are important because some patients with ASBO experience bowel strangulation or ischemia.^2^ Primary management of patients with ASBO by a surgical team reduces healthcare utilization and cost and improves outcomes.^3^ Therefore, prediction of the number of admissions of patients with ASBO could contribute to better outcomes due to the advantages of appropriate management preparation.

With regard to abdominal diseases, the occurrences of appendicitis, cholecystitis, and diverticulitis are more frequent in summer than in winter.^4^, ^5^ In contrast, the winter climate may be associated with a higher incidence of ASBO in Tokyo.^6^ However, seasonal variations in the occurrence of ASBO remain unclear. As fluctuations in barometric pressure were observed at the onset,^7^ we hypothesized that the incidence of ASBO was susceptible to meteorological factors and showed seasonal variation. Japan is located in the mid‐latitudes and has a temperate climate in which the weather is quite seasonal. Therefore, the present study aimed to clarify the impact of seasonal and meteorological factors on the incidence of ASBO using a national inpatient database, and meteorological factors measured in multiple places throughout Japan.

## 2 PATIENTS AND METHODS

### 2.1 Data source

This retrospective study used the Diagnosis Procedure Combination (DPC) database of 42 National University Hospitals. The DPC includes the discharge summary and administrative claim information, including patient demographics, diagnoses, and comorbidities, using the *International Statistical Classification of Diseases and Related Health Problems, 10th Revision* (ICD‐10) codes for surgeries and procedures performed.^8^ This system covers over 1000 hospitals in Japan.

Daily meteorological data were measured using an automated meteorological data acquisition system at each meteorological station in 41 regions in Japan, where the 42 National University Hospitals were located. The mean barometric pressure and temperature readings from continuous measurements (from 0:00 to 24:00) and the cumulative values of daylight hours and precipitation (from 0:00 to 24:00) were regarded as daily weather variables and were obtained from the website of the Japan Meteorological Agency (URL: www.jma.go.jp/jma/index.html). Barometric pressure was estimated based on the altitude of each meteorological station, using the sea level value as a base datum. Day‐to‐day differences in barometric pressure and air temperature were defined as the mean daily variables on the index day minus those on the previous day. Diurnal variation in air temperature was defined as the variation between the highest and the lowest air temperature that occurred during the index day.

### 2.2 Patients

Patients aged 20 years and older at admission, and with unplanned admission precipitating diagnosis of ASBO, were identified between April 1, 2012, and March 31, 2020, in 42 National University Hospitals in Japan. These 42 hospitals were located in 41 cities situated throughout Japan: two hospitals in two cities in the Hokkaido region, four hospitals in four cities in the Tohoku region, five hospitals in four cities in the Kanto region, nine hospitals in nine cities in the Chubu region, five hospitals in five cities in the Kansai region, five hospitals in five cities in the Chugoku region, four hospitals in four cities in the Shikoku region, and eight hospitals in eight cities in the Kyushu region. The diagnosis of ASBO was recorded using the ICD‐10 code K56.5. Patients were excluded for concomitant diagnosis codes of intussusception (K56.1), volvulus (K56.2), peptic ulcers with perforation (K27.1, K27.2, K27.5, K27.6), gastrojejunal ulcers with perforation (K28.1, K28.2, K28.5, K28.6), hernia with obstruction (K40.0, K40.3, K41.0, K41.3, K42.0, K43.0, K43.3, K43.6, K44.0, K45.0, K46.0), gallstone ileus (K56.3), paralytic ileus (K56.0), diverticula of the intestine (K57.X), Meckel's diverticulum (Q43.0), unspecified intestinal obstruction (K56.6), Crohn's disease (K50.X), ulcerative colitis (K51.X), and vascular disorder of the intestine (K55.X). The Charlson Comorbidity Index was calculated using Quan's protocol, and each ICD‐10 code for 12 comorbidities was converted into a score and totaled.^9^

### 2.3 Study design

Eight daily weather variables (mean barometric pressure, mean temperature, mean humidity, daylight hours, precipitation, day‐to‐day differences in mean barometric pressure and mean air temperature, and diurnal variation in air temperature), on a total of 119 802 days (formula: study period [2922 days] ×41 cities), and admission days (days on which patients with ASBO were admitted) were defined. Next, the numbers of monthly admissions of patients with ASBO were compared between each of the 12 months. The relationships between the months and the admission days were evaluated. Further, associations between the admission days and the eight daily weather variables were investigated.

### 2.4 Statistical analysis

Continuous data are presented as descriptive statistics. Continuous data for patient characteristics are presented as medians with interquartile ranges (IQRs). The Kruskal‐Wallis test was used to compare the number of admissions in each of the 12 months of the year. If a significant difference (*P* < .05) was shown, the two‐tailed Mann‐Whitney test with Bonferroni correction for multiple comparisons was used, and *P* < .0042 (as calculated by the formula: 0.05 ÷ 12) was considered statistically significant. The relationships between the months and the days of admission were investigated using logistic regression analysis, in which June acted as the reference category. Multiple logistic regression analyses were also conducted to identify the meteorological factors independently associated with the admission days. The results are presented as odds ratios (ORs) with 95% confidence intervals (CIs). In the logistic regression analysis, statistical significance was set at *P* < .05. Weather variables for each month are presented as the mean and standard deviation. One‐way analysis of variance was used to compare the five weather variables between the 12 months. If a significant difference (*P* < .05) was observed, the two‐tailed unpaired t‐test with Bonferroni correction for multiple comparisons was used, and *P* < .0042 was considered statistically significant. The weather variables on admission days were compared between patients who responded to conservative management and those who needed surgical management using the Mann‐Whitney test. The Statistical Package for Social Sciences version 23.0 (IBM Corp., Armonk, NY, USA) was used for statistical analysis.

## 3 RESULTS

**FIGURE 1:**
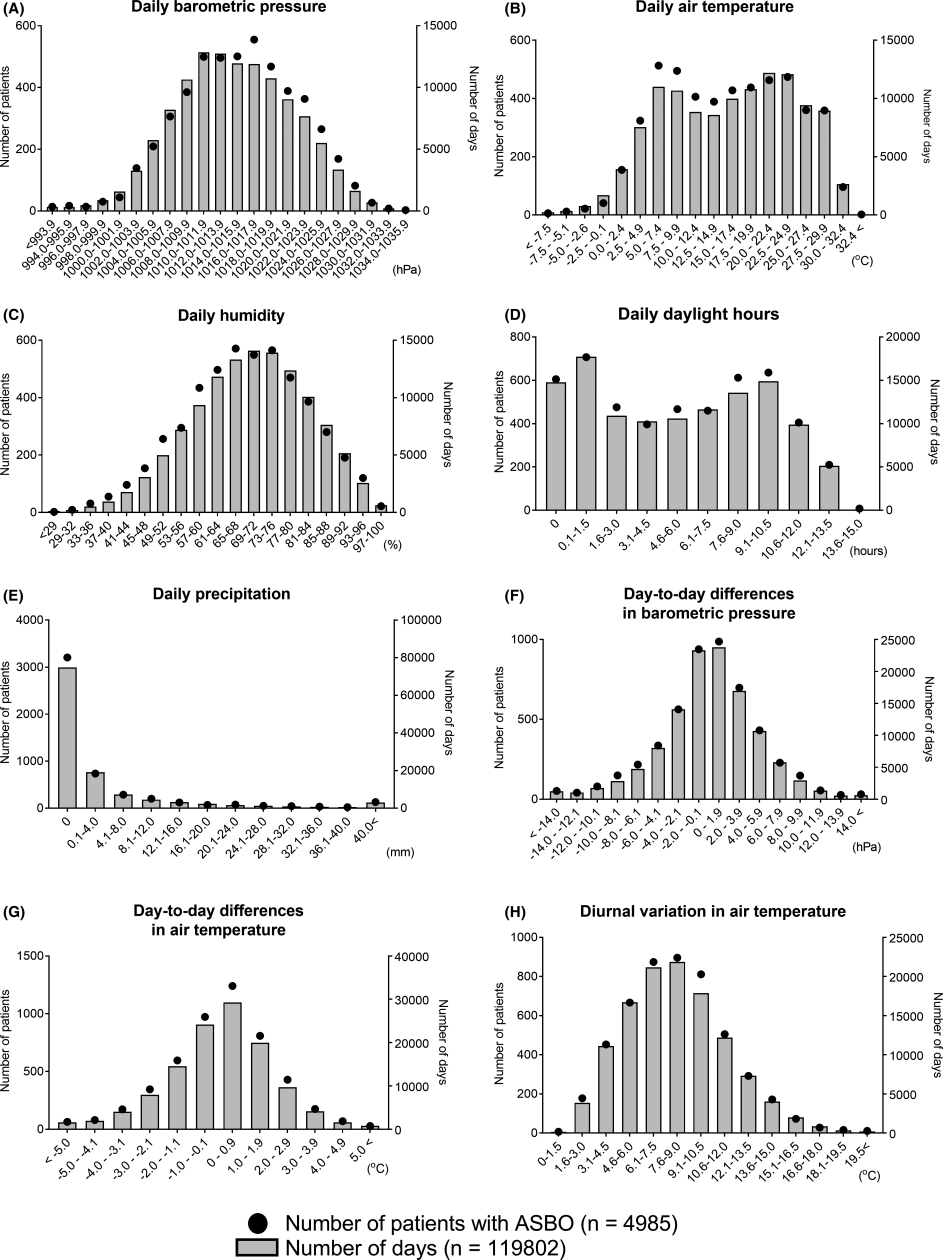
Distributions of the number of patients admitted to hospitals due to adhesive small bowel obstruction (ASBO) (n = 4985) and those of the number of days in all 41 cities (n = 119 802), for each of the eight weather variables (A, barometric pressure; B, air temperature; C, humidity; D, daylight hours; E, precipitation; F, day‐to‐day differences in barometric pressure; G, day‐to‐day differences in air temperature; H, diurnal variation in air temperature). Black circles corresponding to a left Y axis indicate the number of patients with ASBO. Gray bars corresponding to a right Y axis indicate the number of days in all 41 cities

Overall, 4985 ASBO patient admissions were included, and the number of admission days was 4825 days in 41 cities (two admissions of ASBO patients were reported on 60 days). Patient characteristics are shown in Table 1. The median age was 70 years (IQR, 59‐78 years), and 2414 (48.8%) of patients were women. With respect to treatment, 4097 patients (82.2%) responded to conservative management and 888 patients (17.8%) required surgical management. The median length of hospital stay was 11 days (IQR, 8‐18 days). Figure 1 presents the number of patients who were admitted with an ASBO diagnosis and the number of days in the 41 cities. The eight figure parts show the distribution for each weather variable. The shapes of the histograms for the number of patients in the eight variables are similar to those of the distributions of the number of days. However, the number of patients in air temperature and, to a lesser extent, barometric pressure and humidity has two peaks, and the values of weather variables which represent the highest number of patients are different from those which represent the highest number of days in the histograms of these three weather variables.

**TABLE 1:** Patient characteristics

Characteristics	n = 4985
Female, n (%)	2414 (48.4)
Age (years)^a^	70 (59 to 78)
Body mass index (kg/m^2^)^a^	20.4 (17.9 to 22.7)
Barthel index, n (%)	
0	236 (4.7)
5‐60	651 (13.1)
65‐95	839 (16.8)
100	2785 (55.9)
Unknown	374 (8.3)
Charlson comorbidity index, n (%)	
≤2	4485 (90.0)
3	102 (2.0)
≥4	398 (8.0)
Treatment, n (%)	
Conservative management	4097 (82.2)
Surgical management	888 (17.8)
Length of hospital stay (days)^a^	11 (8 to 18)
Daily weather variables on admission days^a^	
Mean barometric pressure (hPa)	1015.4 (1010.3 to 1020.2)
Mean air temperature (℃)	15.7 (8.4 to 22.7)
Mean humidity (%)	69.0 (60.0 to 78.0)
Daylight hours (hours)	5.5 (1.4 to 9.1)
Precipitation (mm)	0 (0 to 2.5)
Day‐to‐day differences in mean barometric pressure (hPa)	0.1 (−2.6 to 2.9)
Day‐to‐day differences in mean air temperature (℃)	0.1 (−1.1 to 1.2)
Diurnal variation in air temperature (℃)	8.1 (6.0 to 10.3)

Abbreviation: hPa, hectopascal.

^a^ Data are presented as median (interquartile range).

**FIGURE 2:**
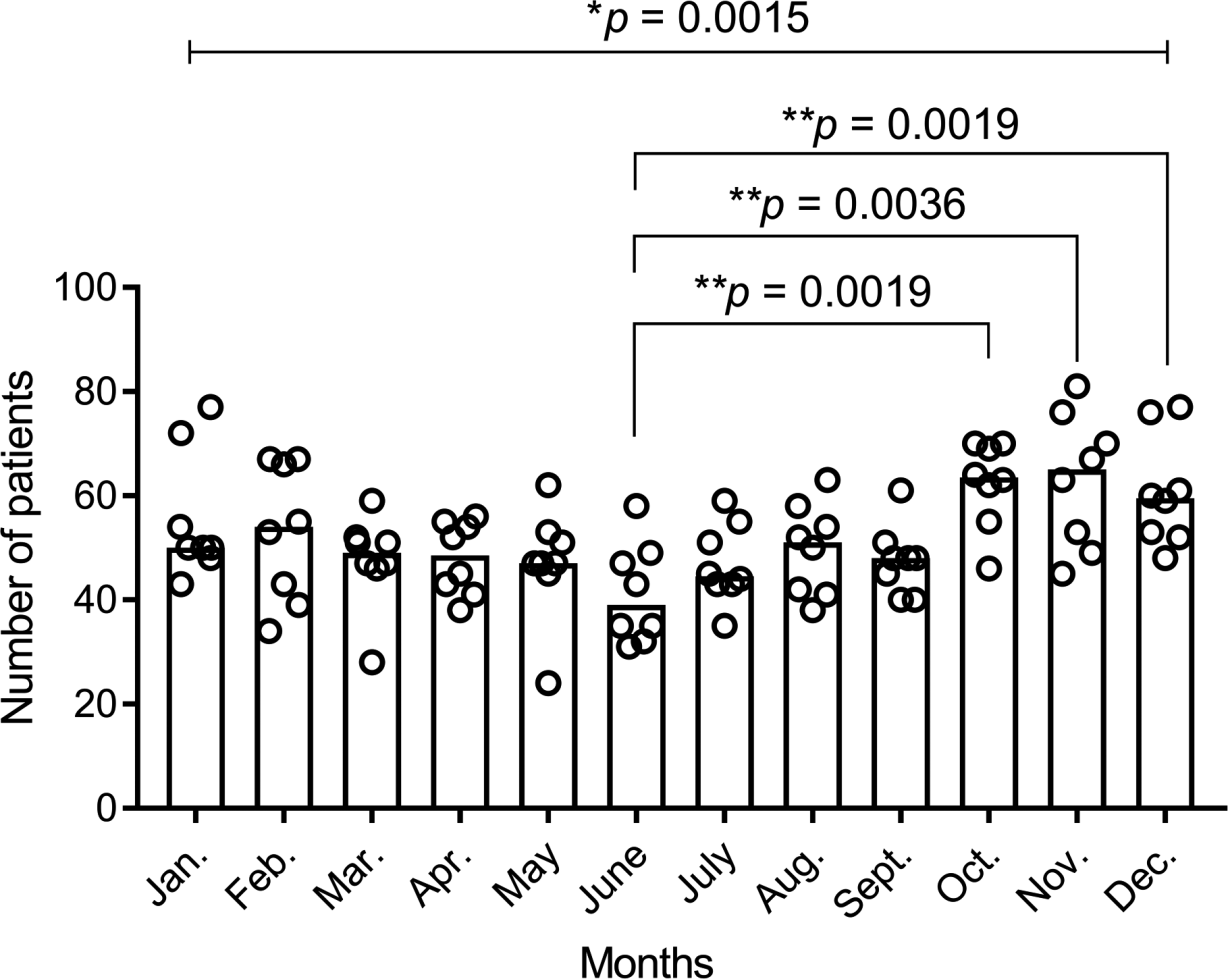
Number of admissions of patients with adhesive small bowel obstruction in each month of the year. Each column has eight circles, which indicate the number of admissions of patients in each month. Data are presented as the median and scatter plot. *P* values with an asterisk were determined by the Kruskal‐Wallis test, and *P* < .05 was considered statistically significant. *P* values with a double asterisk were determined by two‐tailed Mann‐Whitney tests with Bonferroni corrections, and *P* < .0042 was considered statistically significant

The number of admissions of patients with ASBO was significantly different between the 12 months (*P* = .0015). Furthermore, the number of admissions in June each year was significantly smaller than that in October, November, and December (39 [IQR, 32.8‐48.5] vs 63.5 [IQR, 56.8‐69.8], *P* = .0019, 39 [IQR, 32.8‐48.5] vs 65 [IQR, 50‐74.5], *P* = .0036, and 39 [IQR, 32.8‐48.5] vs 59.5 [IQR, 52.3‐72.3], *P* = .0019,) respectively (Figure 2). From the logistic regression analysis, January, October, November, and December were found to be associated with increased risk of admission days compared to June (OR, 1.264; 95% CI, 1.094‐1.460, *P* = .001; OR, 1.454; 95% CI, 1.264‐1.673, *P* < .001; OR, 1.408; 95% CI, 1.221‐1.623, *P* < .001; OR, 1.330; 95% CI, 1.153‐1.534, *P* < .001), respectively (Table 2).

**TABLE 2:** Adjusted association between months and admission days

Variables	Odds ratio	95% CI	*P* value
January	1.264	1.094‐1.460	.001
February	1.146	0.986‐1.332	.077
March	1.043	0.898‐1.213	.581
April	1.141	0.983‐1.323	.063
May	0.993	0.853‐1.156	.929
June	Reference	‐	‐
July	1.037	0.892‐1.206	.633
August	1.117	0.964‐1.296	.142
September	1.144	0.986‐1.327	.076
October	1.454	1.264‐1.673	<.001
November	1.408	1.221‐1.623	<.001
December	1.330	1.153‐1.534	<.001

Abbreviation: CI, confidence interval.

**FIGURE 3:**
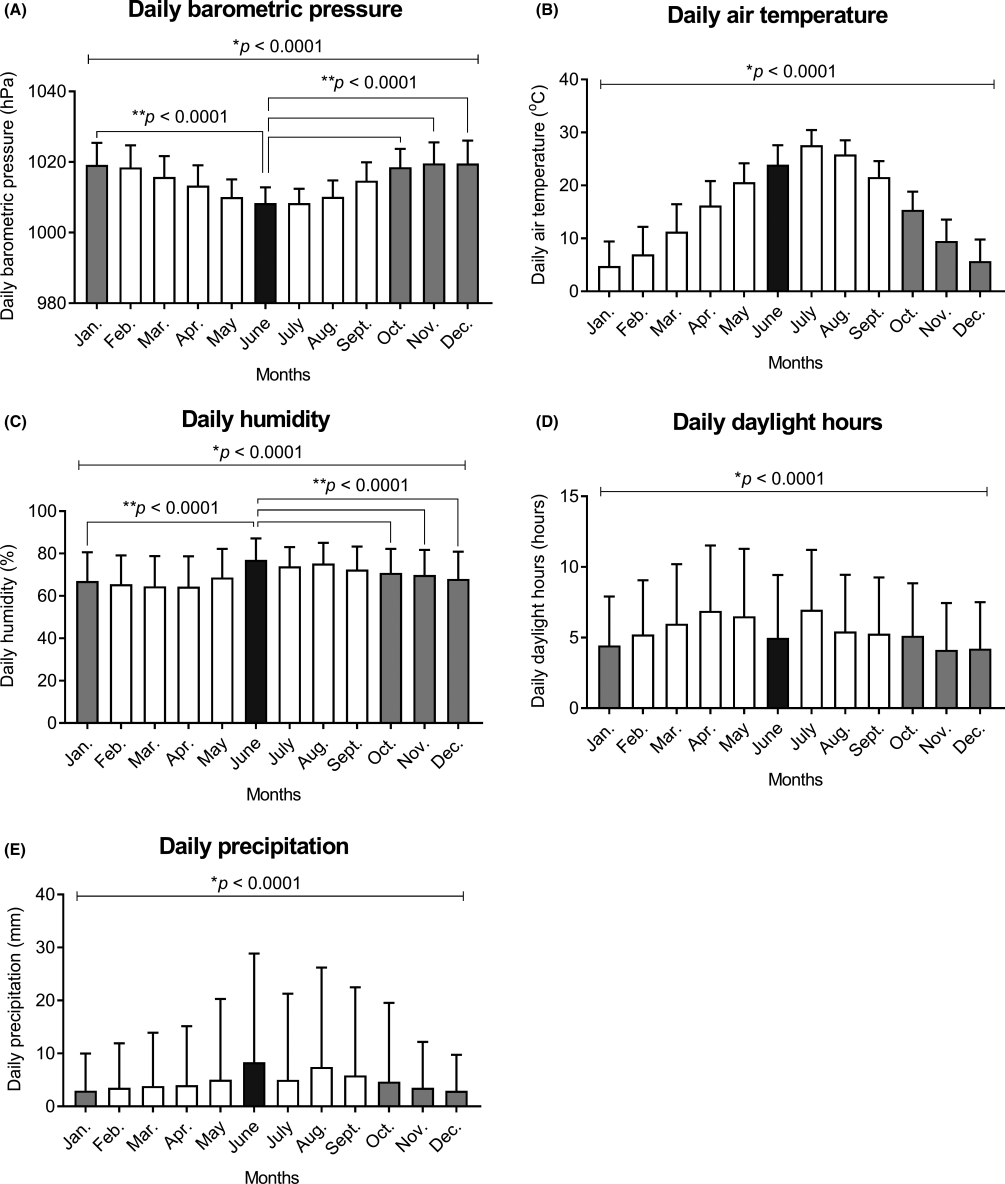
Mean values of five daily weather variables in each month in all 41 regions of Japan during the study period (A, barometric pressure; B, air temperature; C, humidity; D, daylight hours; E, precipitation). Data are presented as mean and standard deviation. *P* values with an asterisk were determined by the one‐way analysis of variance, and *P* < .05 was considered statistically significant. *P* values with a double asterisk were determined by two‐tailed unpaired *t*‐tests with Bonferroni corrections, and *P* < .0042 was considered statistically significant

There was no strong bivariate correlation between the eight weather variables (| *r* | > 0.7 (Table S1). Multiple logistic regression analysis revealed that higher mean barometric pressure and lower mean humidity were associated with an increased risk of admission (OR, 1.009; 95% CI, 1.004‐1.014, *P* < .001; OR, 0.995; 95% CI, 0.993‐0.997, *P* < .001), respectively (Table 3). Daily weather variables in June were characterized by lower barometric pressure and higher humidity than those in January, October, November, and December in Japan (Figure 3) (Table S2).

**TABLE 3:** Multiple logistic regression analysis of admission days

Variables	Univariate			Multivariate		
	Odds ratio	95% CI	*P* value	Odds ratio	95% CI	*P* value
Barometric pressure (hPa)	1.011	1.007‐1.015	<.001	1.009	1.004‐1.014	<.001
Air temperature (℃)	0.996	0.993‐0.999	.013	1.001	0.997‐1.005	.612
Humidity (%)	0.994	0.992‐0.996	<.001	0.995	0.993‐0.997	<.001
Daylight hours (hours)	1.001	0.994‐1.008	.725	‐	‐	‐
Precipitation (mm)	1.000	0.997‐1.002	.679	‐	‐	‐
Day‐to‐day difference in barometric pressure (hPa)	0.998	0.992‐1.003	.403	‐	‐	‐
Day‐to‐day difference in air temperature (℃)	1.004	0.989‐1.019	.613	‐	‐	‐
Diurnal variation in air temperature (℃)	1.006	0.997‐1.015	.178	‐	‐	‐

Abbreviations: CI, confidence interval; hPa, hectopascal.

Lastly, meteorological factors on admission days between patients who responded to conservative management and those who required surgical management were compared (Table S3). There were no differences in these variables between the two groups.

## 4 DISCUSSION

This large‐scale study, using a national inpatient database, confirmed the impact of seasonal and meteorological factors on ASBO in Japan. The number of ASBO admissions in June was smaller than those in October, November, and December. Furthermore, January, October, November, and December were associated with increased risk of the incidence of ASBO compared to June. With regard to weather variables, higher barometric pressure and lower humidity were associated with an increased risk of ASBO admissions. In Japan, June is a rainy season characterized by low barometric pressure and high humidity. These results suggest that seasons and daily weather variables can be helpful for the prevention, early detection, and immediate and appropriate management of ASBO.

Regarding acute surgical diseases in the abdominal organs, seasonal variations were found in appendicitis, cholecystitis, and diverticulitis, indicating that more admissions occurred during the summer months.^4^, ^5^ In contrast, the winter climate has previously been associated with a higher incidence of ASBO in Tokyo.^6^ In accordance with this, the present study revealed that the incidence of ASBO in June was significantly lower than that in December, as well as those in October and November. Furthermore, multiple logistic regression analysis indicated that higher mean barometric pressure and lower mean humidity were associated with an increased risk of admission. In Japan, weather variables in summer were characterized by low barometric pressure and high humidity, whereas those in winter were characterized by high barometric pressure and low humidity (Figure 3). These meteorological data support the seasonal variation in the occurrence of ASBO.

However, the reasons for this variability are unknown because the association between meteorological factors and the etiology of ASBO is unclear. Previously, we reported fluctuations in barometric pressure at the onset of ASBO.^7^ Changes in barometric pressure are also associated with the occurrence of pneumothorax,^10^, ^11^ intracerebral hemorrhage,^12^ myocardial infarction, and brain stroke.^13^ Regarding air temperature, the gastrointestinal tract is innervated by the enteric nervous system, which has cold chemosensors that are activated by low temperature.^14^, ^15^, ^16^ These studies indicate that changes in barometric pressure and air temperature could be associated with the incidence of ASBO. In this study, day‐to‐day differences in mean barometric pressure and mean air temperature, and diurnal variation in air temperature were not associated with the admissions. Further, no weather variable was associated with the need for management (Table S3). Because higher barometric pressure and lower humidity may be surrogate markers of specific meteorological factors that have an impact on the physiological mechanisms underlying the development of ASBO, further research on the etiology of ASBO using detailed meteorological data is needed.

Dietary counseling and nutritional support, including recommendation of a low or modified fiber diet, chewing foods well, and eating smaller amounts of food more often throughout the day, are generally accepted to prevent ASBO in patients who underwent abdominal surgery.^17^, ^18^, ^19^ Our results can strengthen the effectiveness of that advice by means of seasonal and weather variables. In concrete terms, in the period from October to January, and on days of high barometric pressure and low humidity, patients should be careful about the dietary content and eating style to reduce the risk of ASBO, because these seasonal and meteorological factors are associated with the increased incidence. Similarly, these factors can allow healthcare workers to alert patients to avoid developing ASBO but also to prepare to provide prompt and appropriate medical treatment for them.

The key strength of this study is that the data of patients and weather variables were obtained from multiple hospitals and regions. This confirmed its generalizability and external validity. In addition, we used strict inclusion criteria to exclude admissions of patients with non‐adhesive causes of intestinal obstruction, such as hernia and paralytic ileus due to intra‐abdominal inflammation.

This study has some limitations that need to be acknowledged. Firstly, the main limitation was the lack of information on patients’ lifestyles, clinical histories, and conditions. In particular, information about abdominal surgery prior to the incidence of ASBO, such as abdominal diseases and organs, and surgical procedures, was not available due to the nature of the DPC data. Secondly, there are imbalances in the number of patients and admission days among the 41 regions. This could be a selection bias. Thirdly, several cities where the participating National University Hospitals are located do not have meteorological stations. In these cases, meteorological data were obtained from the nearest local meteorological station in the same prefecture (Table S4). Therefore, some differences in the data may have originated from gaps in altitude, longitude, and latitude between these hospitals and the measuring stations concerned.

## 5 CONCLUSION

This large‐scale study elucidated that the incidence of ASBO varies from month to month. In Japan, June weather is characterized by low barometric pressure and high humidity. In contrast, January, October, November, and December weather is characterized by high barometric pressure and low humidity. Weather variables, namely higher barometric pressure and lower humidity, were associated with an increased risk of admissions of ASBO. These results can contribute to the prevention, early detection, and immediate and appropriate management of ASBO.

## DISCLOSURE

Conflict of Interest: The authors declare no conflict of interest for this article.

Ethical Approval: The study protocol was approved by the Institutional Ethical Committee of The University of Tokyo (approval number: 2020388NI), which waived the need for informed consent because of the anonymous nature of the data.

## Supporting information

Table S1

Table S2

Table S3

Table S4

## Data Availability

The data that support the findings of this study are available from the corresponding author, YY, upon reasonable request.
