# Supplementary material for: Impact of seasonal and meteorological factors on the incidence of adhesive small bowel obstruction: A large‐scale study using a national inpatient database
Source: Ann Gastroenterol Surg. 2021 Dec 28;6(4):569–76. doi: 10.1002/ags3.12541 (PMC9271017; doi:10.1002/ags3.12541)
Supplement: Supplementary file 1 — Table S1 [file AGS3-6-569-s004.docx]

**Impact of seasonal and meteorological factors on the incidence of adhesive small bowel obstruction: a large-scale study using a national inpatient database**

| Supplementary Table 1. Pearson correlations between weather variables | | | | | |  |  |  |
| --- | --- | --- | --- | --- | --- | --- | --- | --- |
|  | Barometric pressure | Air temperature | Humidity | Daylight hours | Precipitation | Day-to-day difference in barometric pressure | Day-to-day difference in air temperature | Diurnal variation in air temperature |
| Barometric pressure | 1.000 | -0.528 | -0.268 | 0.110 | -0.258 | 0.357 | 0.020 | 0.139 |
| Air temperature | -0.528 | 1.000 | 0.186 | 0.171 | 0.087 | -0.077 | 0.112 | -0.010 |
| Humidity | -0.268 | 0.186 | 1.000 | -0.581 | 0.406 | -0.325 | -0.046 | -0.483 |
| Daylight hours | 0.110 | 0.171 | -0.581 | 1.000 | -0.357 | 0.304 | 0.197 | 0.635 |
| Precipitation | -0.258 | 0.087 | 0.406 | -0.357 | 1.000 | -0.278 | -0.117 | -0.278 |
| Day-to-day difference in barometric pressure | 0.357 | -0.077 | -0.325 | 0.304 | -0.278 | 1.000 | -0.318 | 0.061 |
| Day-to-day difference in air temperature | 0.020 | 0.112 | -0.046 | 0.197 | -0.117 | -0.318 | 1.000 | 0.327 |
| Diurnal variation in air temperature | 0.139 | -0.010 | -0.483 | 0.635 | -0.278 | 0.061 | 0.327 | 1.000 |
