# Supplementary material for: Impact of seasonal and meteorological factors on the incidence of adhesive small bowel obstruction: A large‐scale study using a national inpatient database
Source: Ann Gastroenterol Surg. 2021 Dec 28;6(4):569–76. doi: 10.1002/ags3.12541 (PMC9271017; doi:10.1002/ags3.12541)
Supplement: Supplementary file 2 — Table S2 [file AGS3-6-569-s001.docx]

**Impact of seasonal and meteorological factors on the incidence of adhesive small bowel obstruction: a large-scale study using a national inpatient database**

| Supplementary Table 2. Mean daily weather variables for the study period in 41 cities | | | | | | | | | | | | |
| --- | --- | --- | --- | --- | --- | --- | --- | --- | --- | --- | --- | --- |
| Variables | January | February | March | April | May | June | July | August | September | October | November | December |
| Barometric pressure (hPa) | 1019 (6.2) | 1018 (6.2) | 1016 (5.9) | 1013 (5.7) | 1010 (5.0) | 1008 (4.4) | 1008 (4.1) | 1010 (4.7) | 1015 (5.2) | 1019 (5.2) | 1020 (5.9) | 1020 (6.5) |
| Air temperature (℃) | 4.7 (4.6) | 7.0 (5.2) | 11.3 (5.2) | 16.2 (4.6) | 20.6 (3.5) | 23.9 (3.6) | 27.6 (2.9) | 25.8 (2.7) | 21.6 (3.0) | 15.4 (3.4) | 9.5 (4.0) | 5.7 (4.1) |
| Humidity (%) | 67.0 (13.6) | 65.5 (13.6) | 64.5 (14.3) | 64.4 (14.3) | 68.8 (13.4) | 77.0 (10.1) | 73.9 (9.1) | 75.3 (9.7) | 72.4 (10.9) | 70.1 (11.3) | 69.9 (11.8) | 68.0 (12.9) |
| Daylight hours (h) | 4.4 (3.5) | 5.2 (3.8) | 6.0 (4.2) | 6.9 (4.6) | 6.5 (4.8) | 5.0 (4.5) | 7.0 (4.2) | 5.4 (4.0) | 5.3 (4.0) | 5.1 (3.7) | 4.1 (3.3) | 4.2 (3.3) |
| Precipitation (mm) | 3.0 (7.0) | 3.5 (8.4) | 3.9 (10.1) | 4.0 (11.1) | 5.1 (15.2) | 8.4 (20.5) | 5.0 (16.3) | 7.4 (18.8) | 5.9 (16.6) | 4.7 (14.9) | 3.5 (8.7) | 3.0 (6.8) |
| Data are presented as mean (standard deviation). | | | | | | | | | | | | |
