# Supplementary material for: Impact of seasonal and meteorological factors on the incidence of adhesive small bowel obstruction: A large‐scale study using a national inpatient database
Source: Ann Gastroenterol Surg. 2021 Dec 28;6(4):569–76. doi: 10.1002/ags3.12541 (PMC9271017; doi:10.1002/ags3.12541)
Supplement: Supplementary file 3 — Table S3 [file AGS3-6-569-s003.docx]

**Impact of seasonal and meteorological factors on the incidence of adhesive small bowel obstruction: a large-scale study using a national inpatient database**

| Supplementary Table 3. Comparison of weather variables between patients who responded to conservative management and needed surgical management | | | |
| --- | --- | --- | --- |
| Variables | Conservative management (n = 4097) | Surgical management (n = 888) | *p* value |
| Barometric pressure (hPa) |  |  |  |
| Median (IQR) | 1015.3 (1010.3 to 1020.2) | 1015.5 (1010.1 to 1020.2) | 0.915 |
| Air temperature (℃) |  |  |  |
| Median (IQR) | 15.6 (8.4 to 22.6) | 16.0 (8.4 to 23.1) | 0.496 |
| Humidity (%) |  |  |  |
| Median (IQR) | 69.0 (60.0 to 78.0) | 70.0 (60.0 to 79.0) | 0.144 |
| Daylight Hours (hours) |  |  |  |
| Median (IQR) | 5.5 (1.4 to 9.1) | 5.6 (1.3 to 9.1) | 0.915 |
| Precipitation (mm) |  |  |  |
| Median (IQR) | 0.0 (0.0 to 2.5) | 0.0 (0.0 to 3.0) | 0.816 |
| Day-to-day difference in barometric pressure (hPa) |  |  |  |
| Median (IQR) | 0.1 (-2.7 to 2.9) | 0.1 (-2.5 to 2.8) | 0.788 |
| Day-to-day difference in air temperature (℃) |  |  |  |
| Median (IQR) | 0.1 (-1.1 to 1.2) | 0.1 (-1.2 to 1.2) | 0.168 |
| Diurnal variation in air temperature (℃) |  |  |  |
| Median (IQR) | 8.1 (6.0 to 10.3) | 8.1 (6.0 to 10.2) | 0.254 |
| hPa, hectopascal; IQR, interquartile range. | | | |
