# Supplementary material for: Impact of seasonal and meteorological factors on the incidence of adhesive small bowel obstruction: A large‐scale study using a national inpatient database
Source: Ann Gastroenterol Surg. 2021 Dec 28;6(4):569–76. doi: 10.1002/ags3.12541 (PMC9271017; doi:10.1002/ags3.12541)
Supplement: Supplementary file 4 — Table S4 [file AGS3-6-569-s002.docx]

**Impact of seasonal and meteorological factors on the incidence of adhesive small bowel obstruction: a large-scale study using a national inpatient database**

| Supplementary Table 4. List of hospital names, prefectures and cities/towns where the referring meteorological stations are located | | | |
| --- | --- | --- | --- |
| Hospital | Prefecture | City/town where the hospital located | City where the reffering Meteorological Station is located |
| Hokkaido University Hospital | Hokkaido | Sapporo | Sapporo |
| Asahikawa Medical University Hospital | Hokkaido | Asahikawa | Asahikawa |
| Hirosaki University Hospital | Aomori | Hirosaki | Aomori |
| Tohoku University Hospital | Miyagi | Sendai | Sendai |
| Akita University Hospital | Akita | Akita | Akita |
| Yamagata University Hospital | Yamagata | Yamagata | Yamagata |
| Tsukuba University Hospital | Ibaraki | Tsukuba | Tsukuba |
| Gunma University Hospital | Gunma | Maebashi | Maebashi |
| Chiba University Hospital | Chiba | Chiba | Chiba |
| Tokyo University Hospital | Tokyo | Tokyo | Tokyo |
| Tokyo Medical and Dental University Hospital | Tokyo | Tokyo | Tokyo |
| Niigata University Hospital | Niigata | Niigata | Niigata |
| Toyama University Hospital | Toyama | Toyama | Toyama |
| Kanazawa University Hospital | Kanazawa | Kanazawa | Kanazawa |
| Fukui University Hospital | Fukui | Eiheiji | Fukui |
| Yamanashi University Hospital | Yamanashi | Chuo | Kofu |
| Shinshu University Hospital | Nagano | Matsumoto | Matsumoto |
| Gifu University Hospital | Gifu | Gifu | Gifu |
| Hamamatsu Medical University Hospital | Hamamatsu | Hamamatsu | Hamamatsu |
| Nagoya University Hospital | Aichi | Nagoya | Nagoya |
| Mie University Hospital | Mie | Tsu | Tsu |
| Shiga Medical University Hospital | Shiga | Otsu | Hikone |
| Kyoto University Hospital | Kyoto | Kyoto | Kyoto |
| Osaka University Hospital | Osaka | Suita | Osaka |
| Kobe University Hospital | Hyogo | Kobe | Kobe |
| Tottori University Hospital | Tottori | Yonago | Yonago |
| Shimane University Hospital | Shimane | Izumo | Matsue |
| Okayama University Hospital | Okayama | Okayama | Okayama |
| Hiroshima University Hospital | Hiroshima | Hiroshima | Hiroshima |
| Yamaguchi University Hospital | Yamaguchi | Ube | Shimonoseki |
| Tokushima University Hospital | Tokushima | Tokushima | Tokushima |
| Kagawa University Hospital | Kagawa | Miki | Takamatsu |
| Ehime University Hospital | Ehime | Toon | Matsuyama |
| Kochi University Hospital | Kochi | Nangoku | Kochi |
| Kyushu University Hospital | Fukuoka | Fukuoka | Fukuoka |
| Saga University Hospital | Saga | Saga | Saga |
| Nagasaki University Hospital | Nagasaki | Nagasaki | Nagasaki |
| Kumamoto University Hospital | Kumamoto | Kumamoto | Kumamoto |
| Oita University Hospital | Oita | Yufu | Oita |
| Miyazaki University Hospital | Miyazaki | Miyazaki | Miyazaki |
| Kagoshima University Hospital | Kagoshima | Kagoshima | Kagoshima |
| Ryukyu University Hospital | Okinawa | Nishihara | Naha |
